# Supplementary figures and images for: The alternative cap-binding complex is required for antiviral defense in vivo
Source: PLoS Pathog. 2019 Dec 19;15(12):e1008155. doi: 10.1371/journal.ppat.1008155 (PMC6946169; doi:10.1371/journal.ppat.1008155)

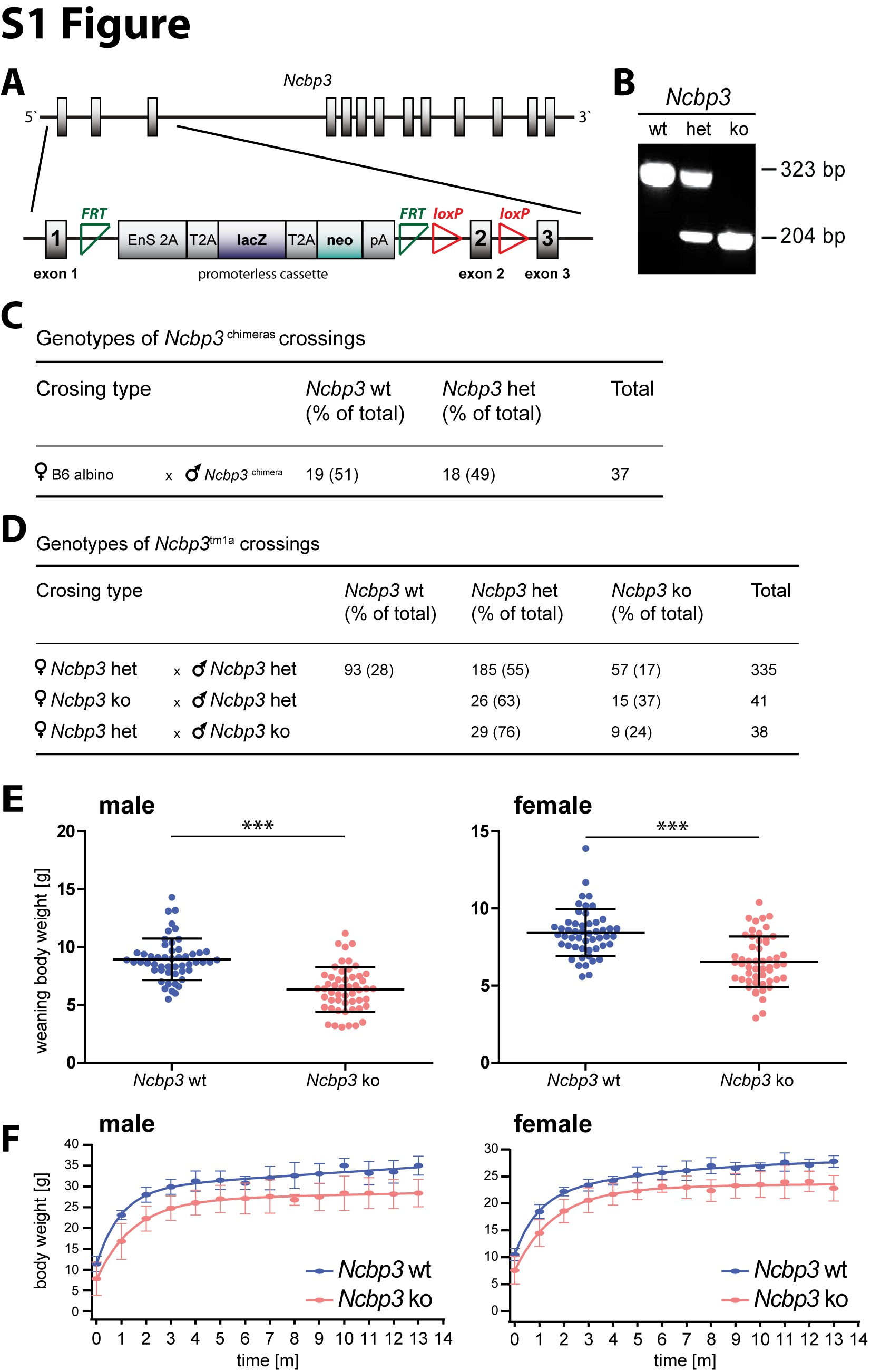

Supplement: S1 Fig — (A) Schematic overview of the promoterless tm1a cassette inserted in intronic Ncbp3 region. The cassette was inserted into the first intron of Ncbp3 gene locus (NM_025818.3) flanked by FRT sites and encodes for a neomycin resistance and a LacZ gene with splice acceptor and a polyA site. Exon 2 is flanked by loxP sites which, after recombination, results in a frame-shift mutation. (B) Genotyping PCR of Ncbp3 tm1a mice. PCR amplification results in a 324 bp construct for Ncbp3 wt mice and a 204 bp construct for Ncbp3 ko (tm1a promoterless cassette insertion) mice. (C) Genotypes of Ncbp3 chimera crossings. Male Ncbp3 chimeras were crossed with C57BL/6 albino and obtained genotypes are represented. (D) Genotypes of Ncbp3 tm1a crossings. Ncbp3 tm1a mice were bred to homogeneity and genotypes obtained for the indicated breeding combination are shown. (E) Body weights of Ncbp3 wt and ko mice at the age of weaning. Body weight of 52 animals per genotype in the age of 19-23 days were monitored. *** P<0.001 as analyzed by one-way analysis of variance (ANOVA) statistics with Bonferroni`s post-test. (F) Body weight development of Ncbp3 wt and ko mice over 13 months. Body weight of 6 animals per genotype and gender were monitored for 13 months. FRT, Flipase Recognition Target; EnS 2A, splice acceptor site; T2A, peptide sequence with self-cleaving function; lacZ, lacZ gene encoding for ß-galactosidase; neo, neomycin resistance gene; pA, simian virus 40 polyadenylation signal; loxP, locus of X-over P1; bp, base pair; wt, wild-type; ko, knockout; Ncbp3, Nuclear cap-binding protein 3. (TIF) [file ppat.1008155.s001.tif]
